# Calcifying Ghost Cell Odontogenic Cyst: Report of a Case and Review of Literature

**DOI:** 10.1155/2011/328743

**Published:** 2011-08-03

**Authors:** Archana Sonone, V. S. Sabane, Rajeev Desai

**Affiliations:** ^1^Department of Oral & Maxillofacial Pathology, C. S. M. S. S. Dental College, Aurangabad, Maharashtra, India; ^2^Department of Oral Pathology and Microbiology, Dr. D. Y. Patil Dental College and Hospital, Pimpri, Pune 18, Maharashtra 400 706, India; ^3^Department of Oral Pathology and Microbiology, Nair Dental College and Hospital, Mumbai, Maharashtra 400 008, India

## Abstract

The calcifying ghost cell odontogenic cyst (CGCOC) was first described by Gorlin et al. in 1962. Calcifying ghost cell odontogenic cyst is comparatively rare in occurrence, constituting about 0.37% to 2.1% of all odontogenic tumors. The most notable features of this pathologic entity are histopathological features which include a cystic lining demonstrating characteristic “Ghost” epithelial cells with a propensity to calcify. In addition, the CGCOC may be associated with other recognized odontogenic tumors, most commonly odontomas. There are variants of CGCOC according to clinical, histopathological, and radiological characteristics. Therefore a proper categorization of the cases is needed for better understanding of the pathogenesis of each variant. Here, we report a classical case of calcifying odontogenic cyst along with a brief review of literature.

## 1. Introduction

Epithelial-lined cysts seldom occur in skeletal bones, because embryonic epithelial rests are normally not found in them. They do, however, occur in the jaws where the majorities are lined by epithelium derived from remnants of the odontogenic apparatus. These odontogenic cysts are classified as either of developmental or inflammatory origin. The calcifying ghost cell odontogenic cyst (CGCOC) is a rare example of a developmental odontogenic cyst, its occurrence constituting about 0.37% to 2.1% of all odontogenic tumors [1]. 

The calcifying ghost cell odontogenic cyst (CGCOC) was first described by Gorlin et al. [7] who were impressed by the significant presence of the so-called “ghost cells.” At that time, they suggested that this cyst may represent the oral counter part of the dermal calcifying epithelioma of Malherbe [2, 3]. Over the years since its first description, it has become clear that the calcifying ghost cell odontogenic cyst (CGCOC) has a number of variants, including features of a benign odontogenic tumor. It was classified as SNOMED code 930/0, in the World Health Organization's (WHO) publication Histological Typing of Odontogenic Tumors [4]. There has been a complete re-evaluation of this lesion by many authors. One major conclusion of Praetorius et al. about this lesion was that, it comprised two entities: a cyst and a neoplasm [2].

## 2. Report of a Case

A 23-year-old female patient reported to our outpatient clinic with the complaint of swelling in upper right side of the jaw that had been present for approximately 2 years. 

**Figure 1:**
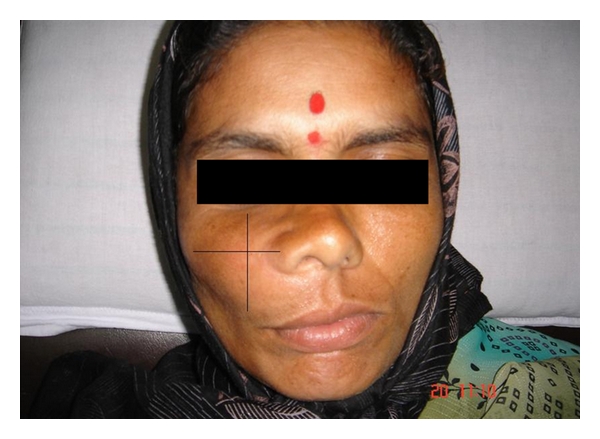
Clinical photographs showing appearance of swelling.

On evaluation, there was an asymmetry involving the right midface region. Swelling was approximately 4 cm × 3 cm in size, extending superoinferiorly from 1 cm below infraorbital rim to angle of the mouth and anteroposteriorly from right ala of the nose to about 3 cm in front of the tragus (Figure 1). Palpation revealed nontender hard bony expansion of the right maxilla. 

**Figure 2:**
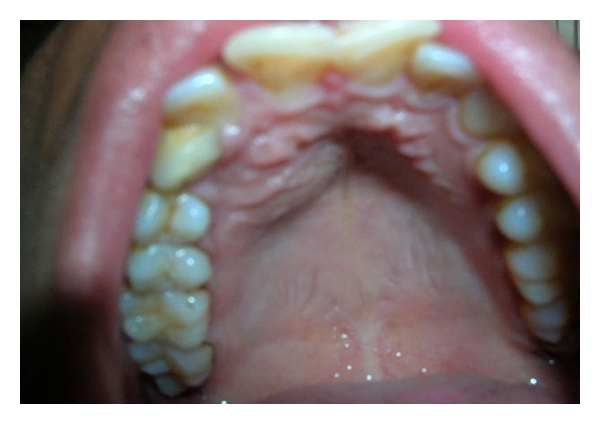
Palatally swelling extending from 11 to 14 regions.

**Figure 3:**
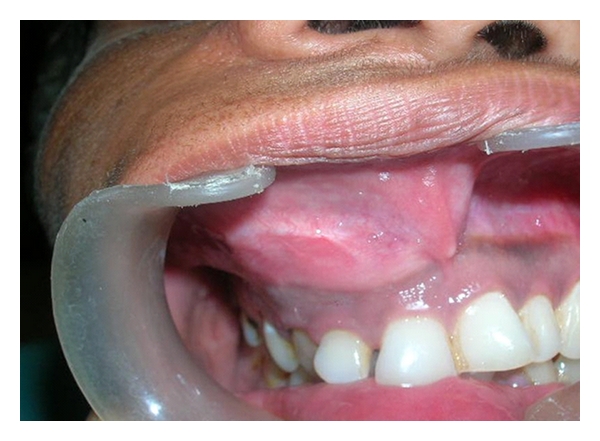
Swelling obliterating buccal vestibule.

 Intraoral examination revealed buccal as well as palatal cortical expansion extending anteroposteriorly from 11 to 14 regions, superoinferiorly obliterating the maxillary vestibule and palatally up to the midpalatine raphe. The mucosa over the lesion was intact (Figures 2 and 3). 

**Figure 4:**
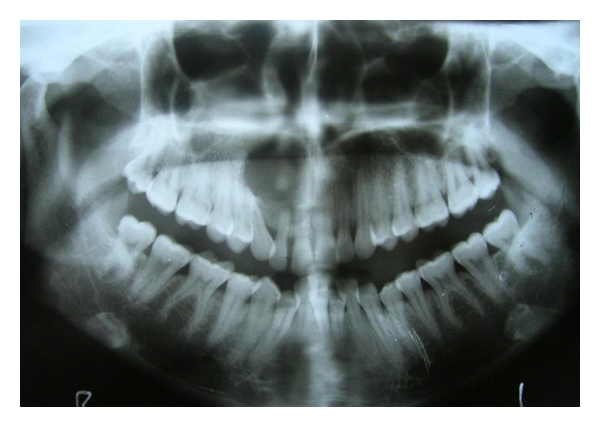
A single calcified material was noted on the orthopentamograph.

**Figure 5:**
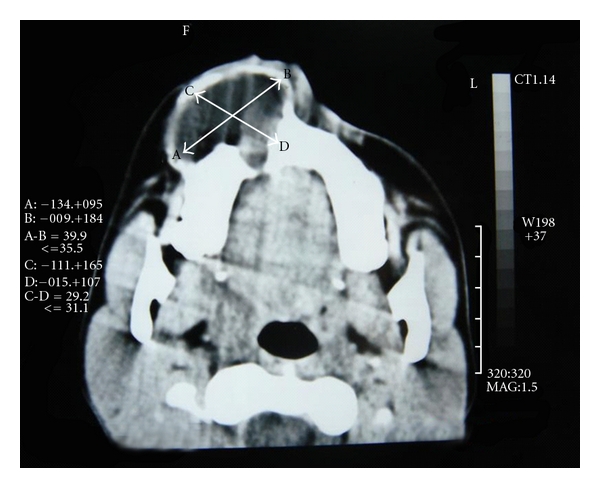
CT showing large expansile lytic lesion arising from the right maxilla extending into the right maxillary antrum and anterior portion of the right nasal cavity.

Radiographic examination disclosed a unilocular well-circumscribed round radiolucency extending from 11 to 15 regions, with radiopaque structures within it (Figures 4 and 5). 

CT scan revealed a large expansile lytic lesion arising from the right maxilla extending into the right maxillary antrum and anterior portion of the right nasal cavity (Figure 5). Based on clinical and radiological findings, differential diagnoses of calcifying odontogenic cyst and calcifying odontogenic epithelial tumor were considered. The adenomatoid odontogenic tumor was not included in differential diagnosis because of the lack of teeth inclusion. FNAC was done, but it was not conclusive. 

The operation was performed under general anaesthesia by enucleation of the lesion, in agreement with the principle of clinical method for treating small cystic lesions of jaws. The enucleated specimen was cystic approximately 5 mm to 4 mm in diameter, entire specimen was sent for histopathological evaluation, and it was reveled as calcifying ghost cell odontogenic cyst.

## 3. Review of Literature

Gorlin and colleagues identified file CGCOC as a distinct pathological entity in 1962 although according to Altini and Farman, the condition had previously been described in German literature in 1932 by Rywkind [5]. It was earlier thought to be an oral presentation of dermal calcifying epithelioma of Malherbe [6, 7]. The CGCOC has also been reported under a variety of other designations including keratinizing cyst [8], keratinizing cyst and calcifying odontogenic cyst (KCOC) [9], calcifying ghost cell odontogenic tumor [10], dentinogenic ghost cell odontogenic tumour, epithelial odontogenic ghost cell tumour, ghost cell cyst, calcifying ghost cell odontogenic tumour, and dentino-ameloblastoma by various authors [11]. The controversy and confusion have existed regarding relationship between nonneoplastic, cystic lesions and solid tumor masses that shares the cellular and histomorphologic features described by authors [12]. In 1971, the WHO described CGOC as a “non-neoplastic” cystic lesion; nevertheless, it decided that the lesion should be classified as a benign odontogenic tumor. In 1992, the World Health Organization (WHO) classified CGOC as a neoplasm rather than a cyst but confirmed most of the cases are nonneoplastic. In view of this duality, many different terminologies have been applied to cystic and solid CGOC variants, but calcifying odontogenic cyst is the preferred term [13]. Different terminologies for CGOC are reviewed in Table 1.

Several classifications of CGOC subtypes have been proposed, but most of them have limitations in separating cystic and neoplastic variant [2]. 

First classification is proposed by Praetorius et al.

**Type 1**

Cystic type:

- simple unicystic type,
- odontoma-producing type,
- ameloblastomatous proliferating type.

**Type 2**

Neoplastic type: dentinogenic ghost cell tumor.

Recent classification suggested classification of CGOC by Reichart [11].

1. Nonneoplastic (simple cystic) variant (CGCOC^a^):
  - with nonproliferative epithelial lining
  - with nonproliferative (or proliferative) epithelial lining associated with odontomas^b^
  - with proliferative epithelial lining
  - with unicystic, plexiform ameloblastomatous proliferation of epithelial lining^c^.
2. Neoplastic variant:
  - benign type (CGCOT^d^):
    - cystic subtype (cystic CGCOT)
      - SMA ex epithelial cyst lining^e^
    - solid subtype (solid CGCOT)
      - Peripheral ameloblastoma-like^f^
      - SMA-like^g^,
  - malignant type (malignant CGCOT or OGCC^h^):
    - cystic subtype,
    - solid subtype.
3. ^
a^Calcifying ghost cell odontogenic cyst.
4. ^
b^Also classified as compound (or complex) cystic ghost cell odontomas.
5. ^
c^Does not completely fulfill the histopathologic criteria of early ameloblastoma as suggested by Vickers and Gorlin.
6. ^
d^Calcifying ghost cell odontogenic tumor.
7. ^
e^With histopathologic features of early ameloblastoma as suggested by Vickers and Gorlin.
8. ^
f^Resembling a peripheral amelobastoma, hence termed peripheral epithelial odontogenic ghost cells tumor.
9. ^
g^Often called central epithelial odontogenic ghost cell tumor.
10. ^
h^Odontogenic ghost cell carcinoma.

CGCOC is a rare developmental cyst. Tomich reviewed about 34 years for odontogenic tumors and cyst at Indiana University School of Dentistry, and he found that only 51 cases of Calcifying ghost cell odontogenic cyst were diagnosed—less than two cases per year! It follows that the average oral and maxillofacial surgeon is likely to see only a case or two during his/her professional career [4]. The odontogenic origin of the CGCOC is widely accepted [1, 14]. The cells responsible for the calcifying odontogenic cyst are dental lamina rests (rests of Serres) within either the soft tissue or bone. Therefore, calcifying ghost cell odontogenic cysts are cysts of primordial origin and are not associated with the crown of an impacted tooth [15]. It most often occurs as a central (intraosseous) lesion [16, 17], whereas peripheral (extraosseous) localization in the soft tissue is rare [18, 19]. 

 There was an almost even gender distribution. In Asians, it showed a higher incidence in younger age group; almost 70% occurred in the second and third decades, whereas in whites, only about 53% occurred in the respective decades, Moreover, in the Asians, the lesions showed a predilection for the maxilla (65%), whereas in whites, the predilection was for the mandible (62%) [20]. The most common site of occurrence has been the anterior part of the jaws. In the mandible, several cases have crossed the midline, but this is less usual in the maxilla [3]. In our case report, the age of female patient was 23 years and occurred at anterior region of maxilla, which was a classical feature for this lesion. 

The central CGCOC (intraosseous) presents as an asymptomatic hard swelling of the jaw that produces expansion than erosion of bone. Pain indicates secondary infection [1]. The clinical features in our case were similar to those described by other authors. The cysts are usually discovered as an incidental radiographic finding. Early in their development, they will appear completely radiolucent. As they mature, they develop calcifications that produce a well-circumscribed, mixed radiolucent-radiopaque appearance. Three general patterns of radiopacity are seen. One is a salt-and-pepper pattern of flecks, the second is a fluffy cloud-like pattern throughout, and the third is a crescent-shaped pattern on one side of the radiolucency in a “new moon”-like configuration [15]. 

In our case report, radiographic examination disclosed a solitary well-circumscribed round radiolucency with single calcified material within it. *X-ray *computed tomography (CT) complements conventional radiographs by depicting the anatomy and topography more accurately. The intra and extraosseous extent of lesions are more accurately determined [1]. In our case, CT scan revealed a large expansile lytic lesion arising from the right maxilla extending into the right maxillary antrum and anterior portion of right nasal cavity.

The definitive diagnosis of CGCOC is made histologically, due to the lesion's lack of characteristic clinical and radiological features, as well as its variable biological behavior. 

**Figure 6:**
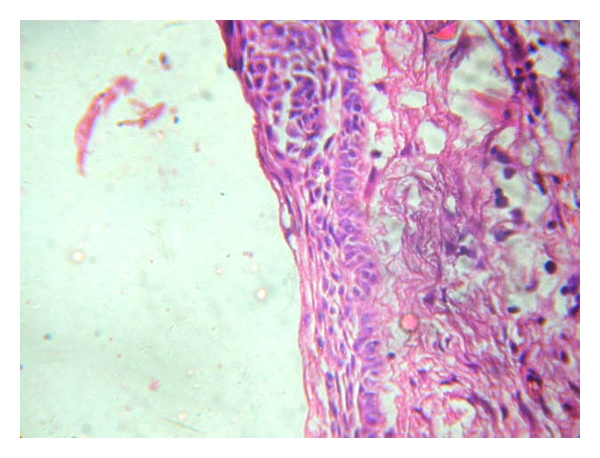
A cystic lumen lined by proliferative odontogenic epithelium (H and E Stained ×100).

**Figure 7:**
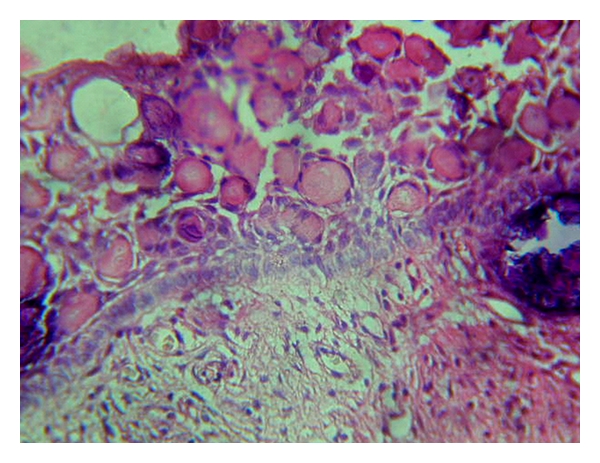
Cystic lumen lined by odontogenic epithelium and areas of “Ghost” epithelial cells projecting into the lumen with areas showing calcification (H and E Stained ×100).

The histological features of a classic calcifying ghost cell odontogenic cyst are characteristic. The microscopical features of lesion showed fibrous capsule with a lining of odontogenic epithelium. The basal layer is made up of ameloblast-like columnar or cuboidal cells and of 4–10 cell thickness. It is overlined by loosely arranged epithelial cells bearing similarity to stellate reticulum of the enamel organ (Figure 6). There are varying numbers of epithelial cells which are devoid of nuclei, eosinophilic, and retaining their basic cell outline (ghost cells). These ghost cells may undergo calcification and lose their cellular outline to form sheet like-area (Figure 7). Many investigators have made effort to clarify the nature of ghost cells by employing special histochemical methods, transmission electron microscopy, and scanning electron microscopy, and various theories have been proposed without any general agreement. Gorlin et al, Ebling and Wagner, Gold, Bhasker, Komiya et al., and Regezi et al. all believed that ghost cells represent normal or abnormal keratinization. Levy suggested that they represent squamous metaplasia with subsequent calcification caused by ischemia. Sedano and Pindborg thought the ghost cells represented different stages of normal and aberrant keratin formation and that they were derived from the metaplastic transformation of odontogenic epithelium. Other investigators suggested or implied that ghost cells may represent the product of abortive enamel matrix in odontogenic epithelium. However, the morphology of ghost cells seems different from that of enamel matrix [14]. Ghost cells are not unique to CGCOC, but are also seen in odontoma, ameloblastoma, craniopharyngioma, and other odontogenic tumors [2]. 

The treatment of cystic lesion involves enucleation with long-terms followup. Recurrence depends on completeness of cyst removal. Prognosis is good for cystic CGCOC and less certain for neoplastic CGCOC [21]. The CGCOC may be associated with other odontogenic tumours such as adenomatoid odontogenic tumor, ameloblastic fibro-odontoma amelobltastic fibroma, and ameloblastoma where the treatment and prognosis in such cases is based on the associated tumors.

## 4. Summary

Our case represents the classical features of calcifying odontogenic cyst, according to Praetorius et al. It comes under category of Type 1(a) simple unicystic type, and according to Reichart, it comes under the category of calcifying ghost cell odontogenic cyst (CGCOC) nonneoplastic (simple cystic) variant with proliferative epithelial lining.

## Figures and Tables

**Table 1:** Terminology of the so-called calcifying odontogenic cyst [13].

Gorlin et al. 1962	Calcifying odontogenic cyst

Gold 1963	Keratinizing calcifying odontogenic cyst (KCOC)

Fejerskov and Krogh 1972	Calcifying ghost cell odontogenic tumor (CGCOT)

Freedman et al. 1975	Cystic calcifying odontogenic tumor (COCT)

Praetorius et al. 1981	Dentinogenic ghost cell tumor (DGCT)*

Ellis and Shmookler 1986	Epithelial odontogenic ghost cell tumor (EOGCT)*

Colmenero et al. 1990	Odontogenic ghost cell tumor (OGCT)*

*These terms are used restrictedly for the solid neoplastic variant of CGOC.
